# Ureteroscopy – an essential modern approach in upper urinary tract diagnosis and treatment

**Published:** 2010-05-25

**Authors:** P Geavlete, M Jecu, B Geavlete, R Multescu, G Nita, D Georgescu

**Affiliations:** Department of Urology ‘Saint John’ Clinical Emergency Hospital, BucharestRomania

**Keywords:** minimally invasive techniques, ureteroscopy, rigid and semi rigid endoscopes, flexible ureteroscopy, upper urinary tract pathologies

## Abstract

In recent years, urology has seen a real explosion in the development of new technologies. Modern treatment techniques replaced classic therapeutic methods, among which open surgery had an important role. Endourologic therapies led to effective and safe interventions, increased patient comfort and reduced costs.

The ‘Saint John’ Emergency Clinical Hospital Department of Urology always intended to align to the new standards of urological treatment including, primarily, minimally invasive techniques, some of them being performed as national premieres.

Ureteroscopy is one of them, thus introducing the rigid and semi rigid endoscopes as part of the therapeutic arsenal of our clinic in 1994 and flexible ureteroscopy in 2002. If the targeted pathology was initially limited to stone disease, ureteroscopy currently covers a wide range of affections, being used both for therapeutic but also for diagnostic purposes.

Thus, the ureteroscopic approach can diagnose and treat a wide range of upper urinary tract pathologies (lithiasis, tumors, malformations, iatrogenic injuries, etc.). The new technology acquisitions made by our clinic, increased performance, resulting in a complete and fast resolution in many cases, previously implying additional effort from the surgeons.

If at first the ureteroscopies' share of daily practice was modest, in recent years it has achieved an extraordinary growth, thus becoming available to both experienced surgeons and young urologists.

We believe that our extensive experience in endourological approach is significant and will have a say in the technological developments, which will help both the patients and the practicing urologists.

Initially limited to stone disease, endourology subsequently found applicability in almost all upper urinary tract pathologies. This fact was mainly due to technical progresses concerning endoscopic equipment as well as to the tendency towards miniaturization providing a variety of instruments from grasping forceps and resection loop to laser fiber. These developments made possible a series of investigative and treatment procedures that were previously accessible only by open surgery and which can presently be performed by endoscopic approach. Thus, ureteroscopy indications and results in the treatment of various diseases were analyzed in long–term studies. From upper urinary tract stones (regardless of location or size) to tumor pathology and from malformations to traumatic or iatrogenic injury, the area of therapeutic approaches is constantly increasing.

The introduction of retrograde ureteroscopy in the diagnostic and therapeutic armamentarium of ‘Saint John’ Clinical Emergency Hospital, Department of Urology, Bucharest, Romania, in 1994, radically changed the investigative and therapeutic protocol in upper urinary tract pathology. The number of procedures performed annually increased from only 45 per year in 1995 to 464 in 2004 and culminated with 783 procedures in 2009. Moreover, the introduction of flexible ureteroscopy (in 2002) allowed the achievement of remarkable progresses in the treatment of upper urinary tract pathology in general and kidney lithiasis in particular.

Between June 1994 and December 2009, retrograde ureteroscopy was performed in a total number of 6053 patients (6202 procedures in total) at ‘Saint John’ Clinical Emergency Hospital, Department of Urology. The patients’ age ranged between 6 and 87 years old. In total, 3451 (57%) patients were men and 2602 (43%) were women. Retrograde flexible ureteroscopy was used in 221 cases, while rigid or semi rigid instruments were used in 5832 patients. Diagnostic ureteroscopic approach was indicated in 859 cases while therapeutic procedures were performed in 5194 patients.

## Diagnostic ureteroscopy

The introduction of flexible ureteroscopes improved the performances of the method by expanding the boundaries of the upper urinary tract accessible for endoscopic inspection. The cases in which imaging provides unclear data benefit nowadays from retrograde uretero–pyeloscopic approach [1].

Diagnostic ureteroscopy was necessary in 859 cases, being applied in order to determine the etiology of hematuria undiagnosed earlier by other methods (412 cases) and of lacunary images after IVP (362 cases), to investigate pathological aspects associated with ureteral duplicity (69 cases) or, in order to evaluate retrograde endopyelotomy results in the selected patients (16 cases).

The success rate of rigid ureteroscopic exploration concerning the detection of the bleeding sources from the upper urinary tract was of 86.9%. The use of flexible equipment allowed its improvement to up to 97.5%.

**Figure 1:**
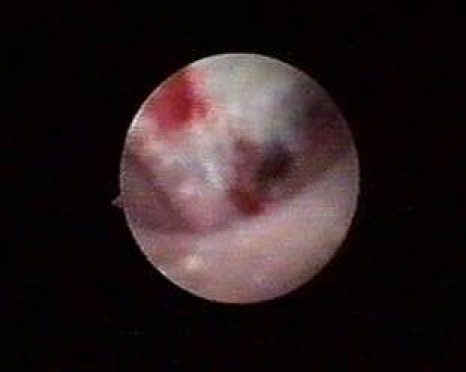
Papillary necrosis

With this regard, the most frequently targeted pathologies highlighted by our experience were represented by tumors of the upper urinary tract, radio–transparent lithiasis, benign urothelial tumors, blood clots, papillary necrosis (Figure 1), and tuberculosis. 

Rigid or flexible retrograde ureteroscopy allowed the detection of lacunary images highlighted by intravenous pyelography (IVP) in 94.1% of the cases.

**Figure 2:**
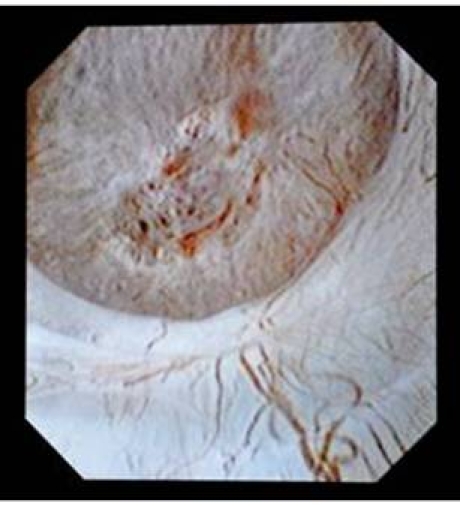
Digital flexible ureteroscopy

The introduction of digital flexible ureteroscopes increased the visibility, thus improving even more the diagnostic accuracy (Figure 2).

**Figure 3:**
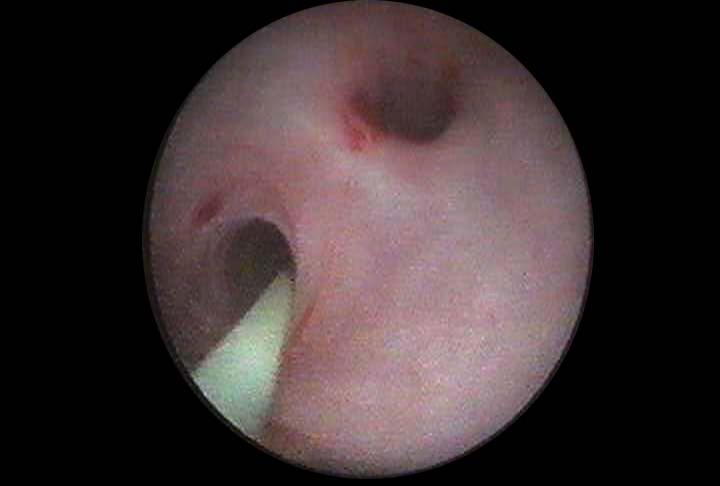
Pyelo–ureteral duplicity stenosis

Pyelo–ureteral duplicity also benefited from ureteroscopic evaluation and/or treatment in the selected cases.  Experience in this field allowed the development of an original classification of pyelo–ureteral duplicity as well as an analysis of the ureters' junction obstruction therapeutic alternatives [2]. The ureteroscopic approach in the treatment of stenosis associated with pyelo-ureteral duplicity was performed in 69 cases (Figure 3). 

## Therapeutic ureteroscopy

Therapeutic ureteroscopic approach was used in 5,191 cases, in ‘Saint John’ Clinical Emergency Hospital, Department of Urology. The indications consisted of reno-ureteral lithiasis (4,452 cases), uretero–pelvic junction stenosis (213 cases), benign ureteral stenosis (62 cases), palliative stenting of extrinsic ureteral stenosis due to neoplastic invasion (259 cases), iatrogenic ureteral injuries (74 cases), resection of ureteral tumors (62 cases), resection of pyelic tumors (6 cases), extraction of ascended stents (56 cases) and ureteroscopic approach of a caliceal diverticula (7 cases). 

### Reno–ureteral lithiasis

Ureteral lithiasis was the main indication of retrograde ureteroscopy. The development of endoscopes and energy sources dramatically improved the performances of this method [3]. In this period, ureteroscopy applied for reno–ureteral lithiasis therapy, which was necessary in 4,452 patients. The mean patients' age was 53 (range 6 to 86 years old).

Single ureteral stones were found in 4,127 patients and multiple stones in 325 patients. Multiple sites of the stones were represented by multiple unilateral ureteral stones (137 cases), ureteral stones associated with ipsilateral pyelic stones (98 cases), concomitant ureteral and contra–lateral pyelic stones (42 cases) and bilateral ureteral stones (48 cases). 

**Figure 4:**
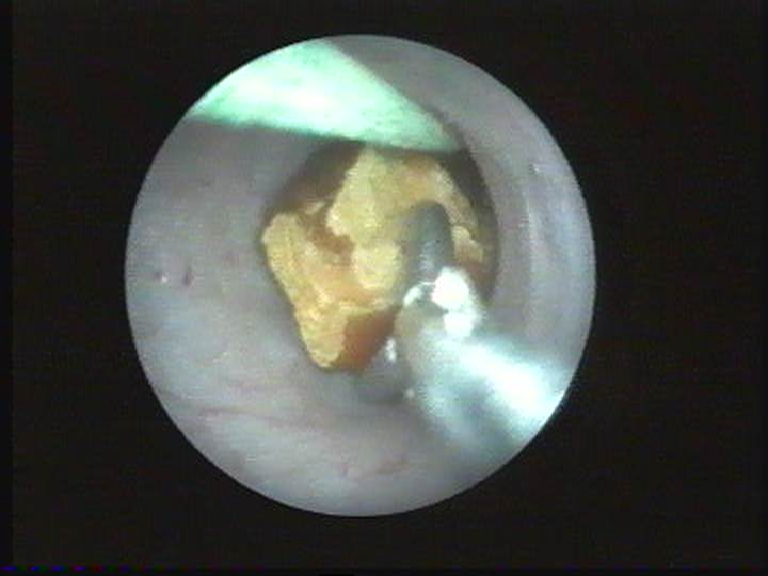
Ureteroscopic approach of ureteral lithiasis

**Figure 5:**
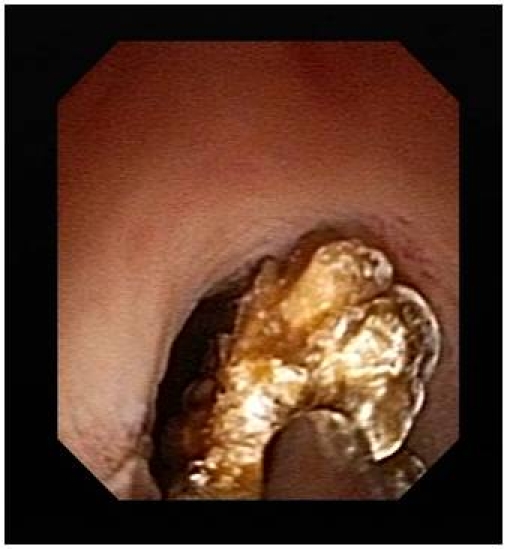
Ureteroscopic approach of ureteral lithiasis (digital ureteroscopy)

In our clinical experience, the treatment of ureteral lithiasis by retrograde ureteroscopy led to an overall ‘stone–free’ rate of 83.5% (Figure 4, Figure 5). In cases of restant lithiasis after ureteroscopy, double J stent indwelling was performed, while complete stone removal was achieved by repeated ureteroscopy or by combining other therapeutic modalities (percutaneous nephrolithotomy – PNL, extracorporeal shock wave lithotripsy – ESWL). Thus, ureteroscopy was repeated in about 7% of cases and the success rate after the second ureteroscopy was of 95%. 

**Figure 6:**
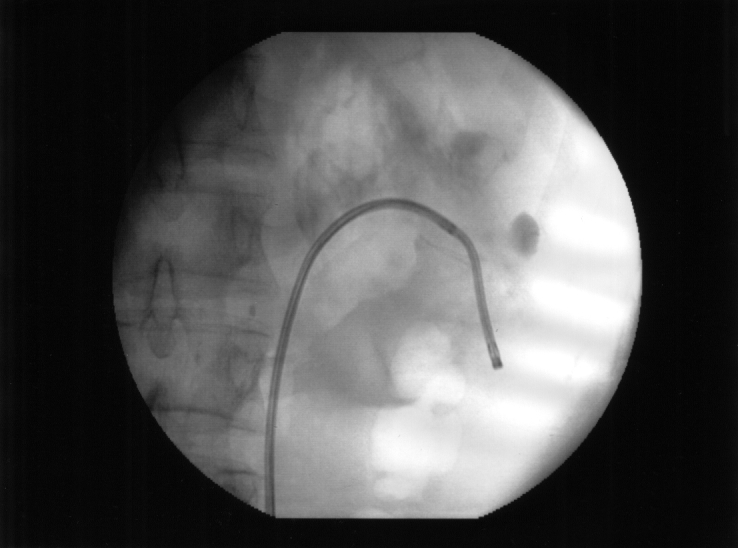
Flexible ureteroscopy (fluoroscopic aspect)

The flexible ureteroscopic approach of pyelo–caliceal lithiasis demonstrated a success rate of 86.1% (Figure 6). For residual calculi, PNL was necessary in 4% of the cases and ESWL was performed in 9.9% of patients.

Emergency ureteroscopic approach in patients with obstructive anuria was performed in 162 patients with obstructive renal lithiasis: 136 cases of ureteral lithiasis in surgical, congenital or functional single kidney, 19 patients with bilateral ureteral lithiasis and 7 patients with ureteral lithiais associated with contra-lateral obstructive pyelic lithiasis. Bilateral ureteroscopy was necessary in 48 cases of bilateral ureteral lithiasis.

**Figure 7:**
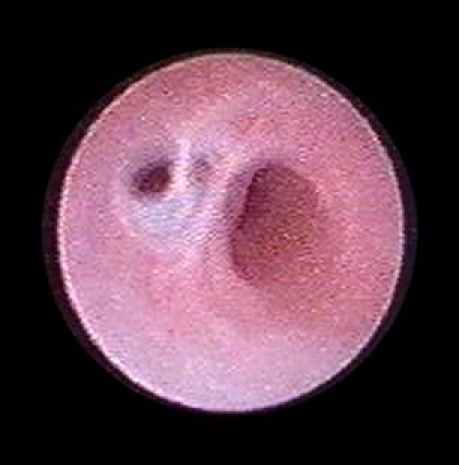
False submucosal path

The rate of intraoperative incidents was of 7.1% while intraoperative complications occurred in 6% of the cases and were represented by minimum injury of the ureteral mucosa, false submucosal paths (Figure 7), ureteral perforation, extra–ureteral migration of the calculi and, exceptionally, ureteral stripping.

The early postoperative complications had an incidence of 10.3% and consisted of ureteral reflux, transient renal colic, persistent hematuria, septic complications (fever, urinary tract infections) and descendant migration of the JJ stent. Late postoperative complications had a reduced incidence and included ureteral stenosis (3 cases) and ureteral reflux (4 cases). The three cases of ureteral postoperative stenosis were recorded at 4, 6 and 12 months, respectively. The treatment consisted of ureteroscopic endoureterotomy and the results were favorable, with no relapses during follow–up. Ureteral reflux therapy consisted of an injection of collagen in the ureteral orifice.

### Uretero–pelvic junction stenosis

The endoscopic retrograde approach in UPJ stenosis was introduced in our country in ‘Saint John’ Clinical Emergency Hospital, Department of Urology, in 1998. 

Retrograde endopyelotomy was performed in 213 patients with UPJ stenosis. Primary stenoses were encountered in 161 cases and secondary stenoses (history of pyelolitotomy and pyeloplasty) in 52 cases. The average length of the UPJ stenosis was of about 1 cm. In 27 cases, the area of stenosis was longer than 2 cm (especially in relapse).

**Figure 8:**
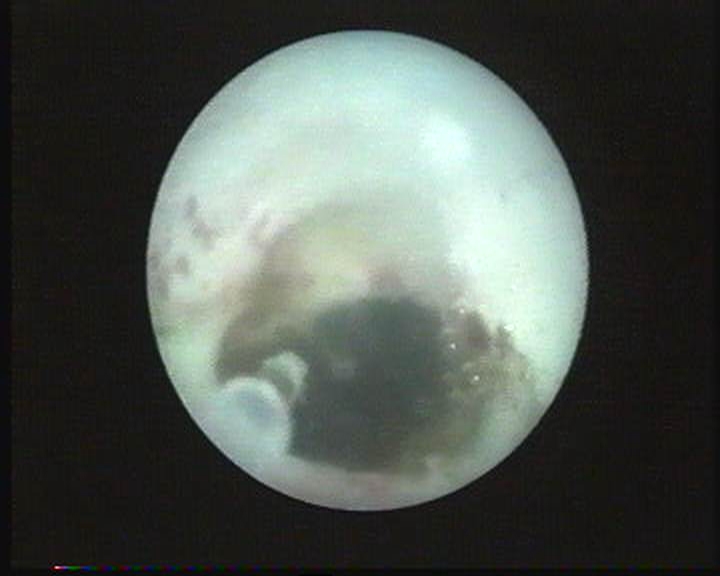
Retrograde endopielotomy using Nd:YAG laser

Cold–knife incision was used in 92 cases and Nd:YAG laser incision in 121 patients (Figure 8). The incision was made all the way through the peripelvic and periureteral fat (or fibrous tissue in cases of secondary stenosis). Extravasation of the contrast medium during the fluoroscopic control confirmed the completeness of the incision. Ureteral stenting was performed for a mean period of 8 weeks. 

The intraoperative complications were minor, without registering significant bleeding or requiring the discontinuation of the intervention. Performing the progressive incision under direct visual control after Doppler ultrasonographic evaluation reduced the risk of damage of any aberrant vessels [4]. 

The evaluation at 3 and 6 months showed the disappearance of the clinically significant symptoms preoperatively described in 90.3% of the patients. IVP showed a large part of the non–obstructive UPJ, which was detected in 198 of the 213 cases, thus significantly reducing the hydronephrosis' degree in 51.7% of cases. In 11 cases, an iterative intervention was required. The 9 and 12 months' postoperative evaluation found a significant reduction of the hydronephrosis degree in 63.4% of patients.

### Inflammatory ureteral stenosis

Retrograde ureteroscopic approach is nowadays considered an alternative in the treatment of inflammatory ureteral stenosis [5].

Since September 1998, cold–knife and Nd:YAG laser endoureterotomy were performed in a total of 62 patients with ureteral stenosis, including 41 men and 21 women. 

The stenosis was located in the distal ureter in 11 cases, in the proximal ureter in 19 cases and in the middle portion of the ureter in 32 cases. The stricture length ranged between 0.5 and 2.5 cm (mean value of 1.3cm.). The intervention was considered effective when the incision penetrated through the periureteral fat. The completeness of the incision was confirmed by the extravasation of the contrast medium confirmed by the fluoroscopic control. Ureteral stenting was maintained for an average period of 60 days. 

Bleeding was minimal in all cases. There were no major intraoperative complications. 

Early and late results of retrograde endoureterotomy were assessed for the entire group of patients at 3, 6, 12, 24 and 36 months postoperatively. Outcomes at 3 months showed the disappearance of the preoperative clinical symptoms in 53 of the 62 patients operated. Doppler ultrasonography, performed in all patients, showed reduced resistivity index in all cases. On IVP, results were materialized by a normal aspect of the ureter in 78.9% of the cases. The results recorded at 3 months were found to remain constant at 6 months postoperatively as proved by the normal IVP aspect of the incised ureter. The average index was 0.6, with values below 0.7 in 52 patients. In more than 60% of the patients, the outcomes at 24 months or more showed the same stable results from the clinical, ultrasonographic and IVP point of view.

### Extrinsic ureteral stenosis due to invading tumors 

Retrograde ureteroscopy was necessary only in cases in which it was not possible to exceed the stenosis area with the JJ stent mounted by cystoscopic approach (248 cases) or in patients in whom Memokath metallic stents indwelling was performed (11 cases). 

Ureteroscopic control allowed the installation of JJ stents in 177 cases. In patients in which stenting could not be achieved not even by ureteroscopic assistance, percutaneous nephrostomy was performed. 

### Iatrogenic ureteral injuries

**Figure 9:**
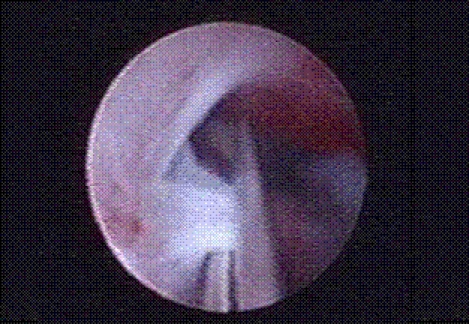
Ureteroscopic cut of the ureteral ligature

Retrograde ureteroscopy was used in 74 cases with iatrogenic ureteral injuries caused by accidental ligature of the ureter during hysterectomy, ureteral wall trauma during open surgery or iatrogenic lesions of the ureteral wall during endoscopic maneuvers. In 40% of the cases of ureteral ligature, the ureteroscopic approach was made within 24 hours, thus making the ureter permeable, followed by ureteral stenting, with subsequent favorable evolution (Figure 9).

In patients with ureteral wall lesions, ureteroscopy and JJ stenting were performed as early interventions after traumatic impairment of the ureter. The success rate was of 87.5%. The failure of endoscopic surgery required open surgery. The long–term evaluation showed favorable results in 53 cases while ureteral stenosis occurred in 7 cases.

### Upper urinary tract tumors

**Figure 10:**
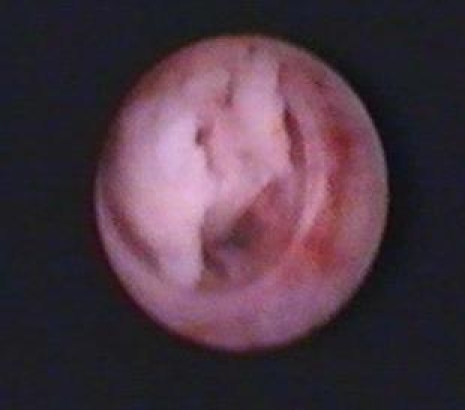
Ureteral tumor

**Figure 11:**
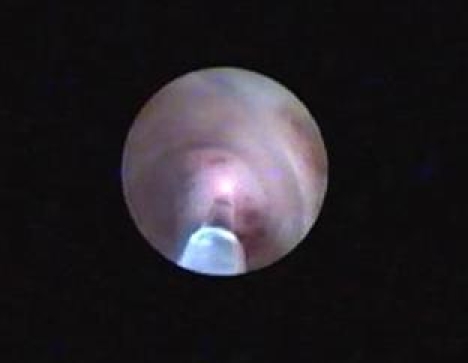
Nd:YAG laser coagulation of ureteral tumor

In our department, the ureteroscopic approach was performed in 62 cases for ureteral tumors and in 6 cases for pyelic tumors (Figure 10). Out of these, 13 were ureteral tumors on a single kidney and 3 cases presented bilateral ureteral tumor. All patients had single superficial tumors with a diameter under 1 cm and a small implantation base. We practiced trans–ureteral resection (34 cases) and Nd: YAG laser coagulation (28 cases) (Figure 11).

Patients were followed by IVP, urinary cytology and ureteroscopy every 3 months. Recurrences were recorded in 42.1% of the patients.

### Ascended ureteral stents

**Figure 12:**
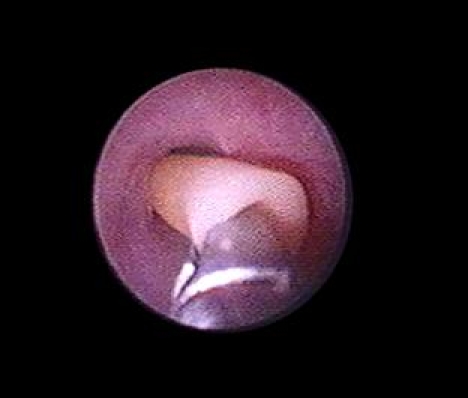
Retrograde ureteroscopy with stent extraction

JJ stent ascension occurred in 56 cases as a postoperative complication of ureteroscopy or other interventions requiring ureteral stenting. In these patients, retrograde ureteroscopy with stent extraction or repositioning was performed (Figure 12).

### Caliceal diverticula approach

**Figure 13:**
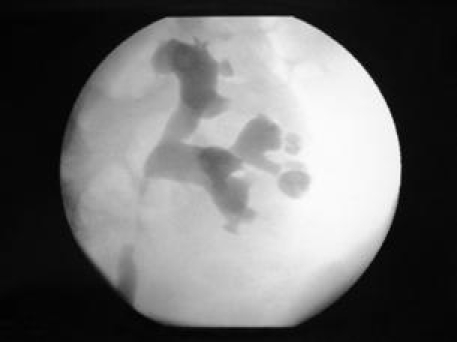
Caliceal diverticula (fluoroscopic aspect)

**Figure 14:**
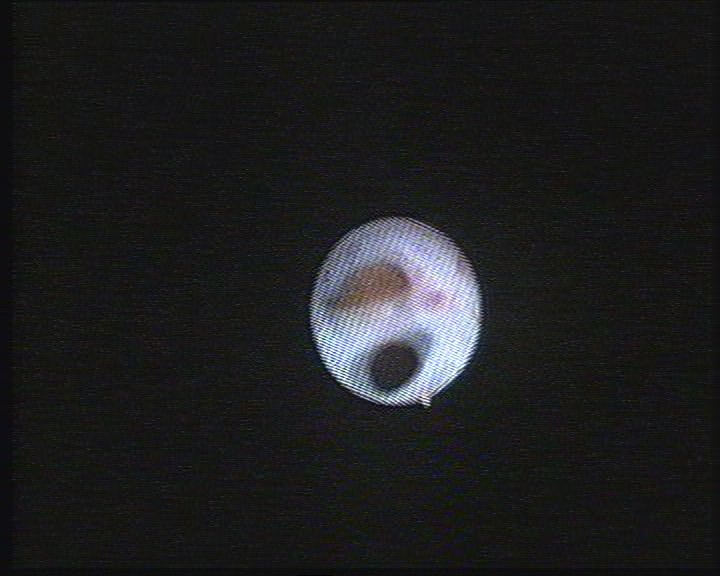
Ureteroscopic approach of caliceal diverticula

Ureteroscopic approach of caliceal diverticula (Figure 13) was applied in 10 cases. The procedure consists in an incision of the diverticular neck and a possible extraction of associated intra-diverticular lithiasis (Figure 14) [6]. This procedure was possible in 7 of 10 cases (70%).

Most failures were related to the lower caliceal location of the stones (2 of 3 cases), the third patient showing a middle caliceal diverticula. The stone–free rate in patients whose diverticula could be approached was of 100%.

## Particular cases

### Retrograde ureteroscopy in children

Continuing our collaboration with the Department of Surgery of ‘M.S. Curie’ Hospital and benefiting from their appropriate equipment, 17 therapeutic retrograde ureteroscopies in children (aged between 6 and 15 years old) were performed in our clinical department. The pathology was represented by ureteral lithiasis (14 cases) and uretero–pelvic junction stenosis (3 cases). For the latter cases, the evolution was excellent, with a remarkably fast return to normal pyelocaliceal system within a mean follow–up period of 17 months. No intraoperative incidents or complications were recorded.

### Retrograde ureteroscopy for lithiasis in pregnant women

The conservative treatment is the first therapeutic option for these cases, due to the fact that most of the stones will be spontaneously eliminated during pregnancy or shortly after birth. The indicated endourologic procedures were represented by retrograde stenting, percutaneous nephrostomy and retrograde ureteroscopy. The safest and least aggressive method is stenting, calculi removal being indicated after birth. This alternative provides remission of symptoms and urinary stasis above the stone, infected or not, with the cost of a minimal morbidity. The effectiveness of stenting was checked in our clinic only by ultrasound control. When stenting was impossible or ineffective, percutaneous nephrostomy was performed. Lately, many authors showed favorable results of retrograde ureteroscopy approach in pelvic ureteral lithiasis in pregnant women [7]. With this regard, the ureteroscopic approach was indicated in 16 cases of pelvic ureteral lithiasis in pregnant women (range 4 to 8 months), in our clinic. There were no intra– or postoperative complications.

## Conclusions

With an experience of more than 6,000 cases ureteroscopically operated in our department by the end of 2009, we believe that we can conclude on some aspects concerning this method. This relatively large number of ureteroscopies may be explained by the wide variety of diseases addressed by these procedures. Many aspects of upper urinary tract pathology now benefit from ureteroscopic approach. 

This minimally invasive technique acknowledged its permanent place in modern healthcare and its results require repeated evaluations. If supporters of ureteroscopy for lithiasis (regardless of the place and size of the calculi) are becoming more numerous, there are still substantial controversies as far as malformations and tumors are concerned, with special concerns for treatment safety and recurrence rate in such cases.

The developments of flexible ureteroscopes (the new digital ones being available since 2007) diversified the indications and improved even more the performances of retrograde approach for upper urinary tract pathology.

Although we strongly support the modern endoscopic diagnostic and treatment approaches while being perfectly aware of the mirage of minimally invasive techniques, we also agree with the fundamental principle stating that any modern method, as seductive and immediately applicable as it may be, should not be implemented with disregard to effectiveness and safety.
